# Prevalence of pneumonia by chest x-ray, associated demographic characteristics and health risk factors among COVID-19 patients in Ghana

**DOI:** 10.4314/gmj.v55i2s.4

**Published:** 2021-06

**Authors:** Joseph A Oliver-Commey, Peter Puplampu, Christian Owoo, Kwame Asare-Boateng, Anita O Yawson, John Tetteh, Benedict N L Calys-Tagoe, Emilia Udofia, Ernest Kenu, Ali Samba, Alfred E Yawson, Margaret Lartey, COVID-19 Collaborators

**Affiliations:** 1 National COVID-19 Treatment Centre, Ga East Municipal Hospital, Ghana Health Service (GHS); 2 LEKMA Hospital, Ghana Health Service; 3 National COVID-19 Case Management Team, Ghana; 4 Department of Medicine and Therapeutics, University of Ghana Medical School, College of Health Sciences, University of Ghana, Accra; 5 Pentecost Convention Centre- National CoOVID-19 Treatment Centre; 6 National COVID-19 Treatment Centre, University of Ghana Medical Centre, Accra; 7 Department of Anaesthesia, University of Ghana Medical School, College of Health Sciences, University of Ghana, Accra; 8 Department of Anaesthesia, Korle-Bu Teaching Hospital, Accra, Ghana; 9 Department of Community Health, University of Ghana Medical School, College of Health Sciences, University of Ghana, Accra; 10 Department of Epidemiology and Disease Control, School of Public Health, University of Ghana; 11 Department of Obstetrics & Gynaecology Department, Korle-Bu Teaching Hospital, Accra, Ghana

**Keywords:** COVID-19, pneumonia, chest X-ray, treatment Centres, Ghana

## Abstract

**Objective:**

The study was conducted to determine the prevalence of radiologically diagnosed pneumonia among COVID-19 patients and associated factors.

**Design, setting, and participants:**

A retrospective manual data extraction of 275 medical records of COVID-19 patients was conducted at two COVID-19 national treatment centres in Accra from March to May 2020. All patients had a chest x-ray done.

**Main outcome and analysis:**

The main outcome was the presence of pneumonia. Descriptive statistics and Chi-square test of independence were employed to determine the associations between independent variables and the presence of pneumonia. All analysis was performed using Stata 16, and a p-value ≤ 0.05 was deemed significant

**Results:**

The prevalence of pneumonia was 44%(95%CI) =38.2–50.0). Chi-square independent test indicated that pneumonia in the COVID-19 patients was associated with educational level, history of domestic and international travel, mass gathering in the past 14 days before diagnosis, and discharge plan (p-value< 0.05). Patients classified as secondary cases (61.5%) and those discharged as fully recovered from the health facility (61.2%) had a higher prevalence of pneumonia. In addition, COVID-19 patients with hypertension (32.1%) and asthma (5.2%) had a significantly higher prevalence of pneumonia.

**Conclusion:**

Overall, the prevalence of pneumonia was 44% and was associated with the demographic and personal characteristics of the patients. Early detection through contact tracing and community surveillance should be intensified to pick up more asymptomatic cases. The role of the chest x-ray for triaging patients and for clinical management of symptomatic patients remains key.

**Funding:**

None declared

## Introduction

The newly emerging COVID-19 disease is a highly infectious disease caused by severe acute respiratory syndrome coronavirus 2 (SARS-CoV-2).

The disease which attacks the lungs and other organs in humans^1^ has been declared a public health emergency of international concern.

In late December 2019, a cluster of patients were diagnosed with pneumonia of an unknown cause in Wuhan, China.^2^ On January 3rd, 2020, the World Health Organization (WHO) was informed about an outbreak of pneumonia of viral origin associated with humans in Wuhan, China.^3^,^4^ International movements have contributed to the rapid spread of the disease globally and have resulted in significant morbidity and mortality. By July 10th, 2020, the pandemic had spread to 213 countries globally and affected more than 12.6 million individuals, with 561,910 reported deaths and 7,318,673 recoveries.^5^ Similarly, in Ghana, a total of 23,463 confirmed cases, 129 deaths, and 18,622 recoveries have been reported during the same period.^5^

The novel SARS-Cov-2 attacks the lower respiratory tract. It produces inflammatory changes consistent with pneumonia, and imaging with ultrasound, chest x-ray or Computer tomographic scan (CT-scan) as an adjunct to polymerase chain reaction test has increased reported cases.^6^,^7^ The main role of imaging in such viral infections is to detect or exclude pneumonia and narrow down other differential diagnoses of pneumonia. This is done using the specific patterns, distribution, and extent of lung abnormalities associated with the potential causative agent.^1^

The clinical features, laboratory and radiological abnormalities seen in COVID-19 are similar to features in other respiratory tract infections.^8^ At the initial stages of the outbreak, case detection was limited to patients with pneumonia. However, as the pandemic evolved, evidence has emerged to indicate other patterns of clinical presentation involving other systems, including the central nervous system, the gastrointestinal and haematopoietic systems. Many infected persons also remain asymptomatic.^9^ Evidence indicates that nearly 69% of patients admitted with COVID-19 have abnormalities on chest x-ray.^10^ As the pandemic evolves, chest x-ray radiographs have been recommended as an essential method of diagnosing COVID-19.^11^ It is now evident that most cases of COVID-19 are asymptomatic or have mild symptoms such as fever cough, dyspnea, myalgia, and fatigue.^6^,^8^ However, it is still recommended that the standard for diagnosing COVID-19 should be viral testing and that chest CT or x-ray should not be adopted as a primary method of diagnosing the COVID-19 disease.^12^ Ghana uses viral testing for diagnosis, and chest CT or x-ray are used for clinical case management. This study was conducted among COVID-19 confirmed cases in the initial stages of the pandemic with two objectives: (i) to assess the prevalence of pneumonia and (ii) to quantify demographic characteristics, symptomatology, and co-morbid factors.

## Methods

### Design and Data Source

Retrospective record review was conducted, and data used for the analysis were manually extracted from the medical records of COVID-19 patients in two national treatment centres in Accra, the Ga East Municipal Hospital and University of Ghana Medical Centre (UGMC), from March when patients were admitted to the health facilities to May 2020.

### Sampling

A total of 256 records from Ga East Municipal Hospital and 19 records from UGMC were reviewed and used for this analysis. Records included for the analysis were from patients whose records showed documentary evidence of the initial positive and the two consecutive negative tests and who underwent a chest radiologic examination on admission (this inclusion criterion was based on the national guidelines prevailing at the time).

### Study population

Upon being diagnosed with a laboratory-confirmed PCR test, COVID-19 patients were sent to the national treatment centres for management. During the period under review, a full recovery from COVID-19 illness in Ghana was based on the WHO standard guide of two consecutive negative laboratory tests from day 14, after being confirmed as positive for COVID-19. Missing data were excluded during the analysis following Strengthening the Reporting of Observational Studies in Epidemiology.^13^ All patients whose data were used in this analysis had recovered after treatment. The analysis involved patients who have recovered either fully in the health facility or were discharged to continue home treatment until full recovery.

### Outcome Variables

The outcome variable in the study was pneumonia which was diagnosed by chest x-ray (classified as yes=1 or no=0). All patients had a chest x-ray done. A case of pneumonia among COVID-19 patients was defined as a patient with a chest xray showing hazy increased lung opacities, consolidation, or pleural effusion, as this is less often reported.^7^,^14^ All chest xrays were taken in posterior-anterior projection at full inspiration as routine or anterior-posterior if supine.^14^

It has been documented that some patients may have normal radiographs in non-severe disease (up to 18%) and fewer in severe disease (3%).^14^ Although thin-section computerized tomography of the thorax has been demonstrably the technique of choice in imaging lung disease^15^. It is relatively more expensive and less widely available. Chest radiographs were more affordable for the majority of patients in this setting. Furthermore, Chen et al. reported bilateral pneumonia as their most common finding on chest radiographs.^16^

### Demographic characteristics and health risk factors

Demographic characteristics included sex (female vs male), age, and educational level (none, primary, secondary and tertiary). Health risk factors in a case of COVID-19 were: domestic travel history within 14 days, international travel history within 14 days, a mass gathering in the past 14 days before diagnosis (no or yes), exposure to similar illness in the past 14 days (no, unknown or yes), case definition [imported, primary, secondary (those who might have acquired it from primary contacts)]; and discharge plan (Full Recovery in the health facility (FR) or Home Treatment Plan (HTP).

### Presenting symptoms

These included a history of fever, sore throat, runny nose, cough, shortness of breath, headache, muscle aches, loss of appetite, and fatigue.

Co-morbid conditions ascertained from clinical records and past medical history or as self-reported by patients were: diabetes mellitus, hypertension, heart disease, asthma, and chronic kidney disease.

### Data analysis

Descriptive statistics were presented as a cross-tabulation of covariates (demographic and health risk variables) by the outcome variable (pneumonia). Chi-square test of independence for all categorical variables (χ^2^) and t-test statistic for a discrete variable (raw age) was applied to summarized data on patients with pneumonia and those without.

### Ethical considerations

Ethical clearance was obtained from the Ghana Health Service Ethics Review Committee (GHS-ERC 006/05/20). Permissions and letters of support were obtained from the heads of the institutions (GEMH and UGMC), where the data were abstracted. Additionally, codes, rather than personal identifiers, were used throughout data abstraction and analysis to ensure anonymity and maintain patient confidentiality.

## Results

The prevalence of pneumonia among the participants was 44%, 95% [8.2, 50.0]. In all, patients aged 30–39 and 50 years and above had the highest proportion of those with pneumonia. Chi-square test of independence indicated a statistically significant association between pneumonia and educational level, domestic travel history within 14 days, international travel history within 14 days, a mass gathering in the past 14 days before diagnosis, case classification, discharge plan and smoking tobacco.

The prevalence of pneumonia was significantly higher among patients with a primary level of education, 41(40.6%), those without domestic, 80(75.5%) and international travel history within 14 days, 119(98.3%). In addition, patients classified as secondary cases, those discharged based on full recovery and those with no history of smoking and alcohol intake had a relatively higher proportion with pneumonia (Table 1).

**Table 1:** Prevalence of pneumonia associated demographic characteristics and health risk factors in COVID-19 patients in Ghana

Demographic characteristics	Pneumonia		Total	χ^2^
	No n(%)	Yes n(%)	N (%)	
	154(56.0)	121(44.0)	275	
**Sex**				0.00
**Female**	70(45.5)	55(45.5)	125(45.5)	
**Male**	84(54.5)	66(54.5)	150(54.5)	
**Total**	154	121	275	
**Age**		41.7±17	39.4±14.0	1.28^Ψ^
**Age group**				6.99
**≤19**	13(8.5)	7(5.8)	20(7.3)	
**20–29**	39(25.3)	29(24.0)	68(24.7)	
**30–39**	24(15.6)	34(28.1)	58(21.1)	
**40–49**	23(14.9)	17(14.0)	40(14.5)	
**50+**	55(35.7)	34(28.1)	89(32.4)	
**Educational level**				31.41***
**None**	7(4.5)	8(7.9)	15(5.9)	
**Primary**	23(14.9)	41(40.6)	64(25.1)	
**Secondary**	39(25.3)	28(27.7)	67(26.3)	
**Tertiary**	85(55.2)	24(23.8)	109(42.7)	
**Total**	154	101	255	
**Domestic travel history within 14 days**				27.08***
**No**	149(96.8)	80(75.5)	229(88.1)	
**Yes**	5(3.2)	26(24.5)	31(11.9)	
**Total**	154	106	260	
**International travel history within 14 days**				100.76***
**No**	62(40.5)	119(98.3)	181(66.1)	
**Yes**	91(59.5)	2(1.7)	93(33.9)	
**Total**	153	121	274	
**Mass gathering in the past 14 days before diagnosis**				5.17*
**No**	138(90.2)	90(97.8)	228(93.1)	
**Yes**	15(9.8)	2(2.2)	17(6.9)	
**Total**	153	92	245	
**Exposure to similar illness in the past 14 days**				0.06
**No**	111(72.5)	65(71.4)	176(72.1)	
**Unknown**	17(11.1)	11(12.1)	28(11.5)	
**Yes**	25(16.3)	15(16.5)	40(16.4)	
**Total**	153	91	244	
**Case classification**				116.07***
**Imported**	91(59.1)	1(0.8)	92(33.7)	
**Primary**	11(7.1)	2(1.7)	13(4.8)	
**Secondary**	52(33.8)	116(97.5)	168(61.5)	
**Total**	154	119	273	
**Discharge plan**				65.14***
**FR**	62(41.1)	91(91.9)	153(61.2)	
**HTP**	89(58.9)	8(8.1)	97(38.8)	
**Total**	151	99	250	
**Illness severity**				0.42
**Asymptomatic**	82(53.2)	60(49.6)	142(51.6)	
**Mild**	64(41.6)	55(45.5)	119(43.3)	
**Moderate/Severe**	8(5.2)	6(5)	14(5.1)	
**Total**	154	121	275	
**Smoking tobacco**				0.17
**No**	131(93.6)	109(94.8)	240(94.1)	
**Yes**	9(6.4)	6(5.2)	15(5.9)	
**Total**	140	115	255	
**Alcohol intake**				2.45
**No**	98(70)	71(60.7)	169(65.8)	
**Yes**	42(30)	46(39.3)	88(34.2)	
**Total**	140	117	257	

NOTE: Ψ represent t-test statistic; Ref denote reference category; P-value notation: *=p-value<0.05; **=p-value<0.01, ***=p-value<0.001

The presenting symptoms of patients with COVID-19 were not associated with the diagnosis of pneumonia (Table 2). Among the co-morbid conditions presented, pneumonia was demonstrated in those with hypertension, 49(40.8%) and asthma, 10(8.8%) (Table 3).

**Table 2:** COVID-19 symptoms and pneumonia in COVID-19 patients, Ghana

Symptoms	Pneumonia		Total	χ^2^
	No n(%)	Yes n(%)	N(%)	
**History of fever**				1.64
**No**	145(94.2)	98(89.9)	243(92.4)	
**Yes**	9(5.8)	11(10.1)	20(7.6)	
**Total**	154	109	263	
**Sore throat**				0.17
**No**	137(89)	96(87.3)	233(88.3)	
**Yes**	17(11)	14(12.7)	31(11.7)	
**Total**	154	110	264	
**Runny nose**				0.34
**No**	146(94.8)	105(96.3)	251(95.4)	
**Yes**	8(5.2)	4(3.7)	12(4.6)	
**Total**	154	109	263	
**Cough**				0
**No**	121(78.6)	86(78.9)	207(78.7)	
**Yes**	33(21.4)	23(21.1)	56(21.3)	
**Total**	154	109	263	
**Shortness of breath**				1.54
**No**	147(95.5)	100(91.7)	247(93.9)	
**Yes**	7(4.5)	9(8.3)	16(6.1)	
**Total**	154	109	263	
**Headache**				2.18
**No**	134(87)	76(80)	210(84.3)	
**Yes**	20(13)	19(20)	39(15.7)	
**Total**	154	95	249	
**Muscle aches**				0.06
**No**	142(92.2)	95(91.3)	237(91.9)	
**Yes**	12(7.8)	9(8.7)	21(8.1)	
**Total**	154	104	258	
**Loss of appetite**				1.69
**No**	144(93.5)	101(97.1)	245(95)	
**Yes**	10(6.5)	3(2.9)	13(5)	
**Total**	154	104	258	
**Fatigue**				0.32
**No**	145(94.2)	84(92.3)	229(93.5)	
**Yes**	9(5.8)	7(7.7)	16(6.5)	
**Total**	154	91	245	

**Table 3:** Comorbidity associated with pneumonia in COVID-19 patients, Ghana

Co-morbidity	Pneumonia		Total	χ^2^
	No n(%)	Yes n(%)	N(%)	
**Diabetes**				2.31
**No**	135(87.7)	110(93.2)	245(90.1)	
**Yes**	19(12.3)	8(6.8)	27(9.9)	
**Total**	154	118	272	
**Hypertension**				7.44**
**No**	115(74.7)	71(59.2)	186(67.9)	
**Yes**	39(25.3)	49(40.8)	88(32.1)	
**Total**	154	120	274	
**Heart disease**				63
**No**	149(96.8)	110(94.8)	259(95.9)	
**Yes**	5(3.2)	6(5.2)	11(4.1)	
**Total**	154	116	270	
**Asthma (requiring medication)**				5.04*
**No**	150(97.4)	104(91.2)	254(94.8)	
**Yes**	4(2.6)	10(8.8)	14(5.2)	
**Total**	154	114	268	
**Chronic kidney disease**				0.72
**No**	153(99.4)	112(98.2)	265(98.9)	
**Yes**	1(0.6)	2(1.8)	3(1.1)	
**Total**	154	114	268	

Overall, hypertension and asthma co-morbid conditions were significantly associated with pneumonia. Lifestyle behaviour such as; alcohol use and non-smokers were found to have a higher proportion of pneumonia, and both were statistically not significant. Presenting symptoms were not associated with pneumonia.

## Discussion

Globally, pneumonia among COVID-19 patients is a common clinical feature in the evolving pandemic.^17^–^18^ The case definition for COVID-19 at the initial stages of the pandemic was mainly patients with diagnosed pneumonia. However, as the pandemic evolved, genomic research and viral testing have enabled diagnoses to be made among asymptomatic persons.^9^ This analysis was conducted primarily to assess the prevalence of radiologically diagnosed pneumonia among persons infected with COVID-19 in Ghana and to relate the context-specific findings to the global symptomatology of the disease.

Overall, the prevalence of pneumonia among the 275 patients with COVID-19 (who had eventually recovered) was 44%. Our analysis revealed that although asymptomatic patients accounted for the highest proportion (142 patients out of 275 patients studied) among persons living COVID-19, pneumonia was relatively high. The proportion of radiologically diagnosed pneumonia was relatively higher among patients with asymptomatic illness (49.5%) than those with mild illness (45.5%) and those with moderate/severe illness( 5.0%). The differences, however, were not statistically significant.

This conforms with Li and colleagues who established^9^ that most patients in China who had successfully recovered from COVID-19 did not only present with pneumonia, they also showed no signs and symptoms (i.e. even at that early stage of the pandemic, asymptomatic persons were living with COVID-19. However, this prevalence (44%) is lower compared to reports in other settings 2,17. In Spain, among a population of pregnant women living with COVID-19, the prevalence of pneumonia was estimated to be over 60%.^18^ Wong and colleagues established a prevalence rate of 69% among COVID-19 infected persons.^10^ The differences in prevalence between the present study and the higher prevalence in other settings could potentially result from different populations characteristics (age and underlying clinical conditions), and the stage of the pandemic in these settings (early stages versus later stages). Another potential explanation for these differences could be that not all symptomatic persons in the present study who underwent chest x-ray had a computerized tomography (CT) scan. A CT scan has a higher sensitivity in detecting pneumonia among patients with respiratory infections.^19^ It can often detect pathology in hidden aspects of a chest radiograph and overcomes the difficulty of shadows imposed by overlapping structures.^15^

Currently, in Ghana, the CT scan is not employed as a screening tool. It is used mainly for patients judged as being severe, in critical condition, or intensive care. However, in developed countries, a CT scan is part of the COVID-19 illness screening process.^1^–^3^,^6^,^10^,^20^

The relatively lower prevalence of radiologically diagnosed pneumonia in these initial COVID-19 patients in the present study might also be attributed in part to the rapid national response to the pandemic. The national response deployed both routine and enhanced surveillance. Healthcare workers promptly identified those with the infection and referred them to the treatment centres for management before the onset of symptoms.

The present study further demonstrated that demographic and health risk factors including educational level, histories of domestic travel, international travel and mass gathering in the past 14 days, case classification, and discharge plan test of proportions were significantly associated with pneumonia in patients with COVID-19. Most of the demographic factors are in congruence with risk factors identified in previous pneumonia studies.^19^,^21^,^22^

Domestic and international travel history and exposure to mass gathering activities with high community-level exposures readily facilitate the transmission of the SARS-CoV-2 virus, especially where observance of preventive measures is lacking. Patients categorized as secondary, i.e. those who might have acquired it from primary contacts, also showed a relatively higher prevalence of pneumonia. This is most likely due to the period of communicability which begins two days before recognizable symptoms.^23^ Therefore, secondary contacts would have been infected before the primary case presents with clinical features of the disease.

Another observation was the relatively higher prevalence of pneumonia in COVID-19 patients with primary level education. It is currently not yet explained whether educational level plays a role in the symptomatology of the disease. Many related factors, however, may play a role. For instance, lower level education may imply lower socio-economic status, poor residential and environmental practices and habits which may increase the risk of exposure. Reduced access to preventive and promotive health information may influence safe practices and required behaviour change. This deserves a further analysis of potential mediating factors in a larger cohort where data collection includes relevant covariates.

Interestingly, a relatively higher proportion of patients without a history of smoking had pneumonia, this though was statistically not significant. The prevalence of smoking in Ghana is relatively low (female and male 2015 age-standardized prevalence: 0.9% and 5.8% respectively)^24^, and the limited exposure offers a potential explanation for the lack of association. This can be explored further on a larger scale.

Underlying co-morbid conditions have been shown to affect the outcome of the COVID-19.^25^ In our analysis, the prevalence of hypertension and asthma among people infected with COVID-19 who had pneumonia was high was significant compared with their counterparts without the condition. It has been established that persons with co-morbid conditions tend to have pneumonia with COVID- 19 infection.^20^,^26^

In this analysis, the prevalence of pneumonia was approximately 41% in those with hypertension and was 9% in those with asthma. The high proportion of pneumonia among those with hypertension in this study is more than twice compared with what was established in Wuhan, China, and more than five times compared with what Chang^27^ and colleagues established in Daegu, South Korea.^28^

### Limitation

The nature of the pandemic and disease transmission did not allow for the routine clinical examination of the chest. Thus, clinical chest examination findings could not be obtained and correlated with the radiological findings. Disease severity could not be directly correlated with the chest x-ray findings as many confounders exist.

## Conclusion

Overall, the prevalence of pneumonia in the 275 COVID-19 patients in Ghana was 44%. Factors associated with pneumonia include educational level, domestic and international travel history, mass gathering, alcohol use, and tobacco smoking. Co-morbid conditions with a significantly higher prevalence of pneumonia in COVID-19 patients were hypertension and asthma. Early detection through contact tracing and community surveillance should be intensified to pick up more asymptomatic cases. The role of the chest x-ray for triaging patients and for clinical management of symptomatic patients remains key.

## Figures and Tables

**Figure 1:**
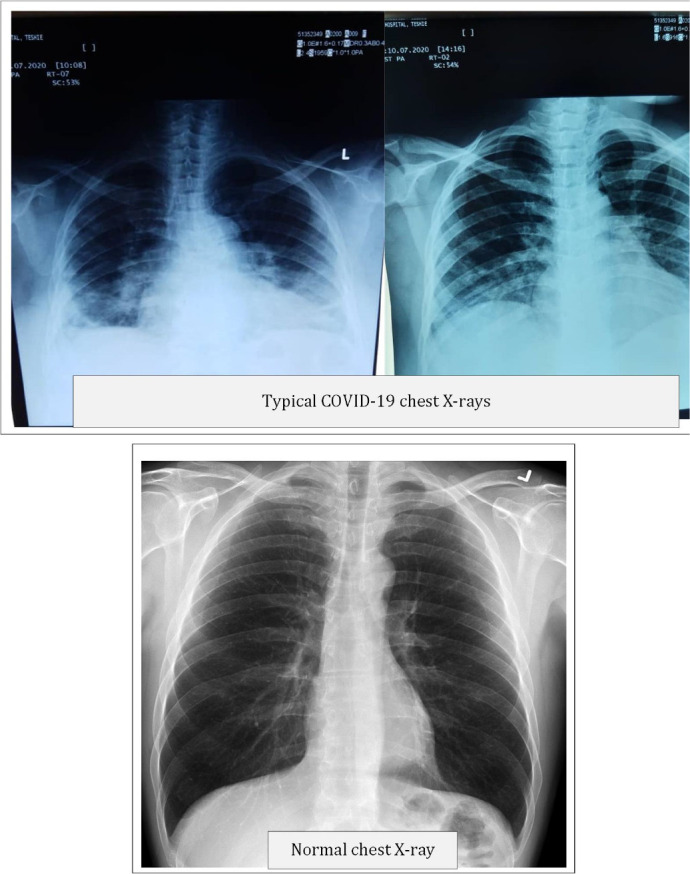
Chest radiographs in COVID-19 pneumonia compared with a normal radiograph
